# Lessons From Iran’s Health-System Response to the June 2025 12-Day Conflict: Implications for Preparedness Amid Continuing Regional Escalation

**DOI:** 10.34172/ijhpm.9419

**Published:** 2026-08-25

**Authors:** Alireza Hajizadeh, Zahra Najafpour, Jalal Saeidpour, Forough Saraee, Edris Kakemam

**Affiliations:** ^1^National Institute for Health Research, Tehran University of Medical Sciences, Tehran, Iran.; ^2^Department of Health Management, Policy and Economics, School of Public Health, Tehran University of Medical Sciences, Tehran, Iran.; ^3^Non-communicable Diseases Research Center, Research Institute for Prevention of Non-communicable Diseases, Qazvin University of Medical Sciences, Qazvin, Iran.

## Introduction

 Armed conflict can disrupt health systems through damage to healthcare facilities, shortages of essential supplies, workforce pressure, population displacement, and interruption of routine services. Experiences from Ukraine and Gaza show that wartime health-system performance depends not only on emergency clinical care, but also on resilient infrastructure, secure supply chains, supported health workers, continuity of essential services, and coordinated governance.^1-3^

 The 12-day conflict that began on June 13, 2025 exposed Iran’s health system to attacks on healthcare-related infrastructure, increased demand for emergency services, workforce mobilisation, and risks to the availability of medicines and medical equipment.^4,5^ Although subsequent regional escalation has altered the broader geopolitical context, the June 2025 conflict remains a clearly bounded case for examining the strengths and vulnerabilities of Iran’s initial health-system response.

 Using the World Health Organization (WHO) Health Emergency and Disaster Risk Management (EDRM) framework, this viewpoint critically examines Iran’s reported health-system response during the June 2025 conflict. It distinguishes announced measures from demonstrated operational capacity, identifies gaps in governance, infrastructure, workforce support, logistics, continuity of essential services, and risk communication, and derives policy lessons relevant to Iran and other health systems exposed to recurrent or prolonged armed conflict.^1^

## Analytical Approach and Scope

 This viewpoint draws on publicly available information from the Iran’s Ministry of Health and Medical Education (MoHME), other national sources, and selected peer-reviewed and international reports. The primary analytical period was June 13–24, 2025; subsequent regional escalation is considered only to contextualize the continuing relevance of the findings.

 Observations were organised using the WHO Health EDRM framework, with emphasis on governance and coordination, health infrastructure, workforce, logistics, continuity of essential services, and risk communication.^1^ Because much of the available evidence consists of government announcements rather than independently verified performance data, the analysis distinguishes reported actions from demonstrated operational outcomes and acknowledges the possibility of reporting bias and incomplete information.

## Emergency Preparedness Context of Iran’s Health System

 MoHME provides national stewardship and coordinates service delivery through universities of medical sciences, supported by a nationwide network of primary care facilities, hospitals, and emergency medical services. This structure enables rapid mobilisation, but preparedness remains constrained by ageing hospital infrastructure, uneven surge capacity, workforce shortages in less-developed areas, and limited interoperability of health-information systems. Although Iran has substantial domestic capacity in medicines and medical equipment, sanctions and procurement restrictions increase the vulnerability of supply chains for specialized products and essential inputs.^6-8^

## Health-System Response During the June 2025 Conflict

 During the conflict, MoHME reported activating national crisis command arrangements and maintaining coordination with hospitals and medical universities. Under health-service continuity and surge capacity, facilities were instructed to maintain readiness, preserve bed capacity, accept casualties, mobilise staff and volunteers, and strengthen referral and emergency services. Under workforce management, leave was cancelled, psychological and social-work teams were deployed, and training materials were issued for psychosocial care and risk communication. Under logistics and supplies, financial resources were allocated for medicines, pharmaceutical reserves were monitored, and blood products, equipment, and essential commodities were managed. Under public health and information management, guidance addressed air pollution, mental health, maternal and child health, public safety, and communication of distressing news, while systems for remote advice and patient-record documentation were expanded. Figure retains the chronological sequence of these reported actions, whereas the text organises them according to the WHO framework.

**Figure:**
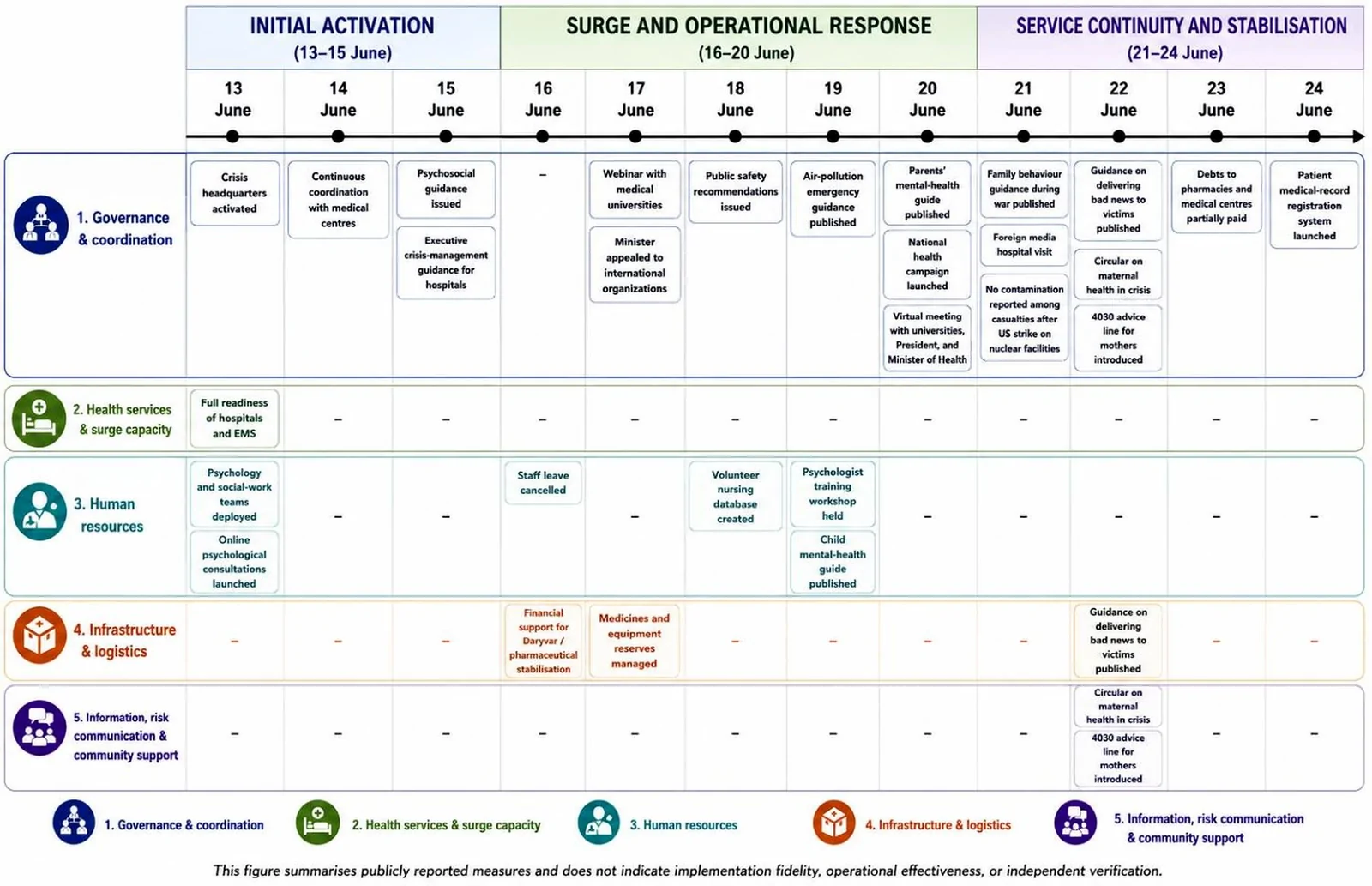
Reported Health-System Response Measures During the June 2025 Conflict, Mapped to WHO Health Emergency and Disaster Risk Management Domains. Note: The figure reflects publicly reported measures and does not indicate implementation fidelity, operational effectiveness, outcome achievement, or independent verification. Dates correspond to the timing of publicly reported actions. Abbreviations: EMS, Emergency medical services; WHO, World Health Organization.

Figure summarises key publicly reported response measures undertaken during 13–24 June 2025 and groups them according to the WHO Health EDRM domains. The figure is intended to illustrate the timing and distribution of reported actions across major preparedness and response areas.

## Critical Appraisal Through the WHO Health EDRM Framework

 Iran’s response demonstrated rapid administrative mobilisation through crisis headquarters, national directives, coordination with medical universities, and measures to maintain service delivery. However, publicly available information does not clarify whether these actions were guided by predefined wartime protocols, activation thresholds, or measurable preparedness standards. Coordination was mainly reflected through meetings and instructions, with limited evidence on interoperability among hospitals, emergency medical services, relief agencies, police, fire services, and passive-defence authorities. Similarly, cancelling staff leave and mobilizing volunteers increased short-term workforce availability but may have intensified fatigue, psychological distress, and safety concerns, highlighting the tension between immediate surge capacity and workforce sustainability.^1,7^

 Hospital readiness, medicine management, public guidance, and expansion of patient-record and remote-advice systems addressed several WHO Health EDRM domains. Nevertheless, ageing infrastructure, limited protective spaces, dependence on electricity and water, sanctions-related supply constraints, and uneven digital connectivity remained important vulnerabilities.^8^ Publicly available reports also provide little information on bed occupancy, referral times, medicine availability, continuity of chronic care, or access among displaced and vulnerable populations. Community engagement largely involved public education and volunteer mobilisation, rather than documented local preparedness mechanisms. Across all domains, the main limitation was the absence of transparent performance indicators, independent verification, and after-action evaluation. Consequently, the reported response demonstrates rapid mobilisation, but does not by itself establish operational effectiveness or sustained health-system resilience.

## Transferable Lessons and International Relevance

 Iran’s experience shares several features with other conflict-affected health systems. In Ukraine, continuity of care depended on organisational flexibility, decentralised service delivery, digital systems, and sustained financing, whereas Gaza illustrates how attacks on facilities, shortages of fuel and medicines, and mass displacement can rapidly overwhelm both emergency and routine care.^2,3^ Iran’s context is distinct because of its centralised health governance, nationwide medical-university network, domestic production capacity, and prolonged exposure to sanctions. These features may support rapid mobilisation, but sanctions and dependence on specialized imports can also intensify vulnerabilities in logistics, equipment maintenance, and access to essential medicines.^7^

 The main transferable lesson is that short-term mobilisation cannot substitute for sustained preparedness. Countries facing war, sanctions, or recurrent crises require predefined command arrangements, protected and decentralised service capacity, redundant supply and communication systems, workforce protection, and explicit plans for maintaining chronic, maternal, mental-health, and primary-care services. Public reporting should also include measurable operational indicators rather than announcements alone. Thus, the Iranian case is internationally relevant not as a model to be replicated, but as evidence that resilience depends on converting emergency mobilisation into institutionalized preparedness, independently evaluated performance, and continuous learning.

## Policy Priorities and Implementation Considerations

 Policy priorities should directly address the gaps identified above. First, Iran should formalize intersectoral emergency command, define activation thresholds, and conduct regular mass-casualty and continuity-of-operations exercises. Second, hospital resilience should be strengthened through protected spaces, backup electricity and water, evacuation plans, and decentralised referral capacity. Third, strategic stockpiles and alternative procurement routes are needed for medicines, blood products, oxygen, equipment, and specialized inputs, particularly where sanctions increase supply-chain vulnerability.^1,7^ Workforce measures should combine reserve staffing and rapid mobilisation with protected rest periods, occupational safety, psychosocial support, and compensation. Preparedness plans should also protect chronic, maternal, mental-health, primary-care, and public-health services rather than focusing only on trauma care.

 Implementation should be phased according to feasibility, affordability, acceptability, and infrastructure readiness. High-cost facility upgrades may require risk-based prioritisation, while training, stock monitoring, referral protocols, and after-action reviews are comparatively feasible short-term measures. Telehealth can support continuity of care, but dependence on electricity, internet access, digital literacy, interoperable records, and secure communication limits its reliability during conflict; offline records, telephone-based services, radio communication, and local referral alternatives are therefore necessary. International organisations and humanitarian actors can support technical assessment, independent monitoring, workforce training, emergency supplies, and protected humanitarian procurement channels. Their involvement should complement—not replace—national capacity and should be governed by transparent coordination mechanisms that preserve equitable access despite sanctions and political constraints.^1,7,9^

## Limitations

 This viewpoint relies primarily on publicly available information and MOHME reports, which may be affected by selective reporting and incomplete disclosure. Independent data, operational indicators, and outcome measures were largely unavailable; therefore, the effectiveness of reported actions could not be directly assessed. The analysis also cannot fully determine whether announced policies were consistently implemented across facilities or translated into sustained operational capacity. In addition, evidence from subsequent regional escalation remains limited, restricting assessment of whether lessons from the June 2025 conflict were incorporated into later preparedness and response. Accordingly, the findings should be interpreted as a structured appraisal of reported measures rather than a definitive evaluation of health-system performance.

## Conclusion

 Iran’s response to the June 2025 conflict showed that rapid mobilisation can preserve essential health functions during a short-term emergency, but it does not by itself demonstrate sustained preparedness or resilience. The principal weaknesses were limited transparency on operational performance, uncertainty about implementation across facilities, vulnerabilities in workforce protection, infrastructure, supply chains, and continuity of routine care, and the absence of independent evaluation. The main policy message is therefore the need to convert emergency actions into institutionalized, measurable, and continuously evaluated preparedness capacities. This lesson extends beyond Iran: health systems exposed to war, sanctions, or recurrent crises require resilient governance, protected service delivery, redundant logistics and information systems, and mechanisms for institutional learning before the next emergency occurs.

## Disclosure of artificial intelligence (AI) use

 The authors would like to disclose that, to improve the consistency and clarity of the manuscript and to draw the figure, an AI tool (ie, ChatGPT) was used.

## Ethical issues

 Not applicable.

## Conflicts of interest

 Authors declare that they have no conflicts of interest.
